# Investigation of Real-Time Photorepair Activity on DNA via Surface Plasmon Resonance

**DOI:** 10.1371/journal.pone.0044392

**Published:** 2012-08-29

**Authors:** Rıza Kizilel, Enis Demir, Selimcan Azizoglu, Hande Asımgi, Ibrahim Halil Kavakli, Seda Kizilel

**Affiliations:** 1 Chemical and Biological Engineering, Koc University, Sariyer, Istanbul, Turkey; 2 Material Science and Engineering, Koc University, Sariyer, Istanbul, Turkey; 3 Molecular Biology and Genetics, Koc University, Sariyer, Istanbul, Turkey; St. Georges University of London, United Kingdom

## Abstract

The cyclobutane pyrimidine dimer (CPD) and 6–4 lesion formations along with the specific breaks on strands are the most common type of DNA damage caused by Ultraviolet light (UV) irradiation. CPD photolyase I and II construct two subfamilies of flavoproteins, which have recognition and repair capabilities of CPD sites on both single stranded (ssDNA) and double stranded DNA (dsDNA) with the aid of blue light energy. The other types of flavoprotein family consist of cryptochromes (CRY) that act as photoreceptors in plants, or circadian rhythm regulators in animals. Recent findings showed that a specific type of Cryptochrome-*Drosophila*, *Arabidopsis*, *Synechocystis*, Human (CRY-DASH) has photorepair activity on ssDNA. In this work, real-time interactions between CRY-DASH and ss/dsDNA as well as the interactions between *Vibrio cholerae* photolyase (VcPHR) and ss/dsDNA were investigated using Surface Plasmon Resonance (SPR). The interactions were then characterized and compared in order to investigate the effect of different types of flavoprotein on UV damaged ss/dsDNA. SPR results confirm the specific binding of VcPHR and CRY-DASH with UV treated DNA. This study is the first instance to quantify the interactions of UV treated and untreated DNA with flavoproteins.

## Introduction

UV is an electromagnetic radiation with a wavelength in the range of 10–400 nm which can cause mutagenic effects by converting a pyrimidine base on DNA to an excited state. [1] The excited base is then capable of reacting with other molecules to form covalently linked stable photoproducts. [1] CPD and pyrimidine (6–4) pyrimidone photoproducts ((6–4)PPs) are produced as a result of formation of stable photoproducts between adjacent pyrimidines within the same DNA strand. These four base-ring photoproducts can be lethal to cell metabolism as they may block the DNA/RNA polymerases on the same DNA strand or inhibit polymerase progression during both DNA replication or transcription. [2]–[4]

One of the repair mechanisms to prevent DNA damage caused by the UV radiation from sun light at 290 nm to 320 nm is photorepairing. It is a three-step process where an enzyme called photolyase bounds to the pyrimidine dimers of DNA at dark. The chromophores in DNA photolyase emerging in archaea, eubacteria, and eukaryotes utilize light in order to eliminate pyrimidine dimers from DNA lesion by catalyzing the cleavage of the cyclobutane ring of the pyrimidine dimer. [5], [6] During the last step of the repair mechanism, the photolyase protein dissociate from DNA. [7], [8] Cryptochromes, on the other hand, share sequence similarity to photolyases and are known to be the enzymes with no DNA repair activity. Further studies have shown that CRY regulates growth and development in plants and the circadian clock in animals. [9]–[11] Despite their DNA-binding capability with single-stranded DNA(ssDNA), they are introduced as photoreceptors, as they are deficient in the repair activity for CPDs in double-stranded DNA (dsDNA). [7], [12], [13]

The more recently discovered subclass of the cryptochrome/photolyase family, called CRY-DASH, is found in cyanobacteria, eubacteria, and vertebrates. [14], [15] The crystal structure of CRY-DASH is determined for *Synechocystis* CRY-DASH [16] and *Arabidopsis thaliana cry3*, [17] and the overall protein folds are found to be similar to class I CPD photolyases. [18], [19] The structural similarity is mostly based on an N-terminal α/β-domain and a C-terminal α-domain with the flavin adenine dinucleotides (FAD) cofactor inside a U-shaped conformation. Similar to photolyases, FAD is fully reduced to FADH^-^ form (catalytically active form) during photoactivation. [20] The surface features around the FAD-binding pocket of cry3 and *Synechocystis* CRY-DASH have been found to be essential for DNA binding as in DNA photolyase. [21] Also, it has been shown that energy transfer from 5,10-methylenetetrahydrofolate (MTHF) to FAD in CRY-DASH, which indicates that energy transfer in CRY-DASH shares mechanistic similarities between photolyase that repairs damaged DNA and CRY-DASH that repairs ssDNA. [22] Despite their similarity at both structural and amino acid levels, CRY-DASH proteins lack C-terminal extensions which are thought to give them the signaling activity. [23] However, it was shown that DASH cryptochromes repair CPDs specifically in single-stranded DNA (ssDNA), [24] and therefore there has been emerging necessity for classification of DASH type cryptochromes as ssDNA-specific photolyases.

In previous studies, photorepair activity of repair proteins have been investigated using various assays such as transformation, absorption, restriction site restoration, and enzyme sensitive site assays. [25] These assays can only give information about the kinetics of repair activity within minutes to hours with excessive required labeling steps. In this study, we were particularly interested in determining the real time interaction of photolyase/cryptochrome family in the repair of UV-irradiated and UV undamaged ssDNA and dsDNA using SPR. It is an excellent method to monitor changes in the refractive index near the vicinity of modified surfaces. The event of capturing the analyte (protein) by the ligand (ssDNA/dsDNA) gives rise to a measurable signal, and therefore, monitors the changes between interacting molecules in real time. Their features such as label-free detection, real time data analysis, and ease of use make them well suited tool to quantify biomolecular interaction. [26] Our results demonstrated that the binding constant for the interaction of VcPHR and UV damaged ssDNA is within the 0.8–1.8 nM range, where the binding constant for the interaction of VcPHR and UV damaged dsDNA is within the 18.53–26.08 nM range. These values are consistent, and have the same order of magnitudes with the interaction constants found in previous biochemical assays for UV damaged ssDNA and dsDNA. [27], [28] To our knowledge, this study is the first instance for the observation of the activity of photolyase proteins with ss/ds DNA using SPR.

## Materials and Methods

### Apparatus and Reagents

For all experiments, two-channel SPR device SensiQ Discovery and BioCap sensor chips (neutravidin modified surface) were used (ICx Nomadics, Oklohoma City, USA). All experiments were conducted at a flow rate of 50 µL/min and at 25°C. The buffer reagent, 1X phosphate buffer saline tablets, sodium hydroxide pellets and Tween® 20 were purchased from Sigma Aldrich (Steinheim, Germany). EDTA was purchased from Applichem (Darmstadt, Germany). Dodecyl sulfate sodium salt (SDS) was bought from Merck (Hohenbrunn, GE). Synthetic oligonucleotides were purchased from The Midland Certified Reagent Co. (Midland, TX, USA). The base sequence of the 5′- Biotinylated probes (48-mers) were 5′- (Biotin) - GACGCAGATCTACGAATTCGCTTAATTCGCTGCACCGGATCCCGCTAG-3′. In order to make double stranded DNA substrate, non biotinylated complementary strand was used for hybridization. Immobilization buffer was prepared from one PBS tablet in 200 mL dH_2_O (10 mM phosphate buffer, 2.7 mM KCl, 137 mM NaCl, pH 7.4). Tween® 20 (0.005% (v/v))was also used in the running buffers as a surfactant to prevent the non-specific protein binding to sensor surface. NaOH (100 mM) and 0.1% (v/v) SDS were used as regeneration buffer and as instrument cleaning solution.

**Figure 1:**
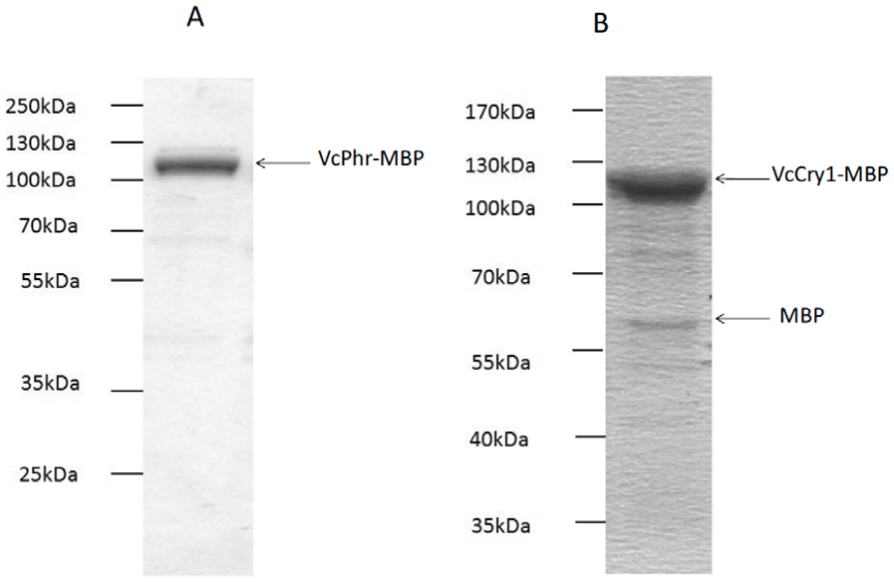
SDS PAGE analysis of purified A; VcPhr1-MBP B; VcCry1-MBP. Proteins were expressed in UNC523 photolyase deficient *E.coli* cells and purified by affinity chromatography using amylose resin. Purified VcCry1 (2 µg) of and purified VcPhr (5 µg) were analyzed on %10 SDS-PAGE and stained with by Coomassie blue. Protein ladders shown in the figure were PageRuler plus prestained protein ladder (Fermentas) for VcCry1.

### Preparation of Wild Type *Vibrio Cholerae* Photolyase *(*VcPHR), Wild Type *Vibrio Cholerae* Class I Cryptochrome (VcCRYI)

VcPHR and VcCry1 were prepared using previously established method by Worthington et al. [29] The plasmids encoding pUNC2001 (MBP-VcPhr), and pUNC2002 (MBP-VcCry1) were kindly obtained from Prof Aziz Sancar (University of North Carolina-Chapel Hill). To express proteins, VcPhr and VcCry1 genes in pmalc2x bacterial expression vector were transferred into *E.coli* UNC 523 (phr-, uvrA-). The proteins were overexpressed by adding 300 µM isopropyl-1-thio-b-D-galactopyranoside (IPTG) until OD_600_ was reached to 0.6–0.8. The cultures were shaken to grow for another five hours at room temperature after IPTG induction. Next, the proteins were purified through affinity chromatography using amylose resin according to manufacturer’s protocol (New England Biolabs, Inc.). Overexpression and purity of proteins were analyzed by Coomassie blue staining on 10% SDS-PAGE.

**Figure 2:**
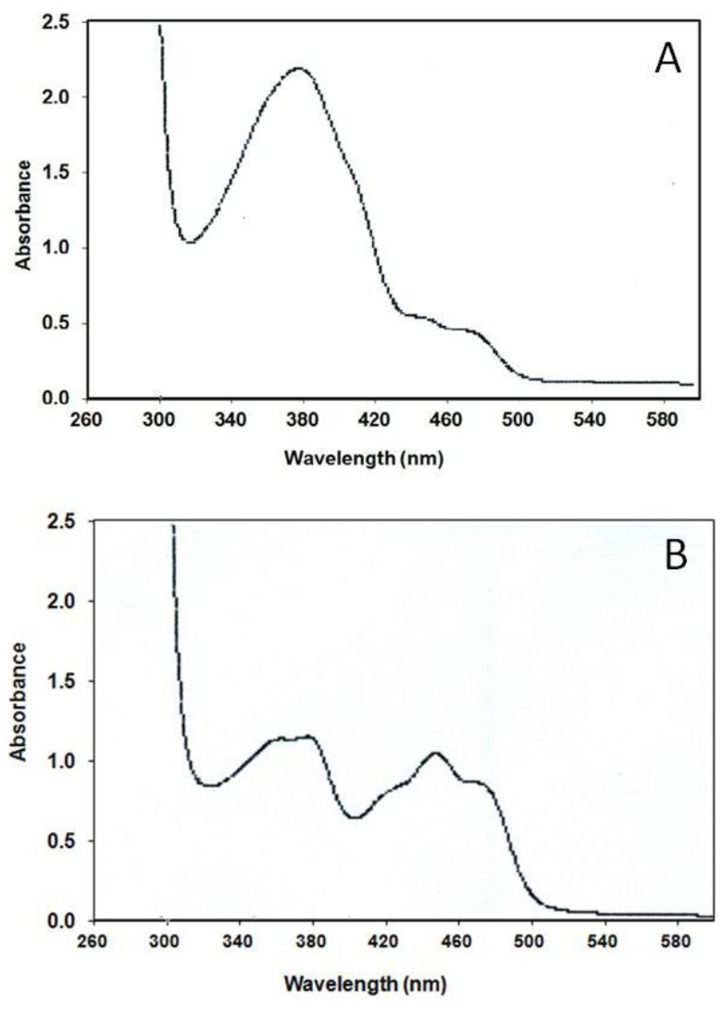
Absorption spectra of purified proteins. Absorption spectra of purified proteins were taken between 260–600 nm wavelengths. A; VcCry1-MBP. MTHF absorption at 380 nm and the minor peaks between 420–480 nm were the indication of different oxidation states of FAD in this purification. B; VcPhr-MBP. The absorption peaks at 380, 420, 440 and 480 nm shows reduced, oxidized, neutral radical forms of FAD existence in the purified VcPhr.

### UV-Irradiation of DNA Substrate and the Assessment of UV Lesions on DNA Substrate

Single stranded 48 bp oligonucleotides containing one T-T sites in the middle was suspended in sterile distilled water at final concentration of 160 µM as a stock suspension and appropriate dilutions were made in PBS buffer for SPR experiments. Stock DNA substrate was irradiated with Slyvania G8W at a fluence rate of 0.5 J/m^2^s with total fluence of 30 J/m^2^ by Slyvania G8W. The method used for UV damaged substrate was applied as described elsewehere. [30] The UV fluencies were measured using a UVX digital radiometer (Ultraviolet Products Inc.) with the appropriate sensor detecting UV light at 254 nm. Also, in order to prepare UV damaged DNA substrate, the 48 bp single strand DNA was irradiated by UV light with a flux of 0.5 j/m^2^.s for 6 minutes to saturate damaged DNA with lesions, and the amount of damage was monitored by the decrease in UV absorbance due to UV lesion formation at 260 nm. [24] The total fluency of UV light irradiation was 175 J/m^2^. After 100 J/m^2^ irradiation, most of single stranded DNA substrates were damaged by UV irradiation and the whole DNA sample was saturated with UV lesions. Since the most commonly occurring lesions produced by UV light[1]–[3], [24], [31] are CPD’s, we decided to use 30 j/m^2^ which was also described in previous studies. [4], [29] The ssDNA substrate prepared for this study carried mostly CPDs which was in sufficient amount for VcCry and VcPHR to bind and repair in SPR experiments. The UV damage formation was assessed by the decrease in absorption of DNA substrate at 260 nm over time with a Nanodrop ND-1000 spectrophotometer Nanodrop, Wilmington, DE (Figure S1). [24]

**Figure 3:**
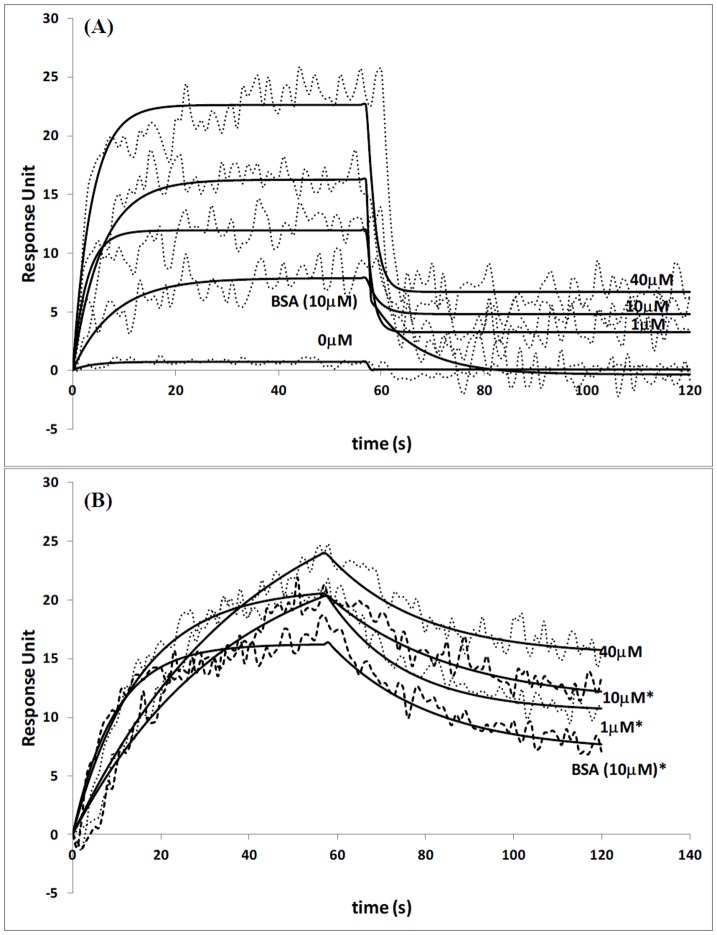
(A) Interactions of VcPHR and undamaged ssDNA at various protein concentrations. Injection concentrations of 0, 1, 10 and 40 µM were used for VcPHR, while for BSA 10 µM injection concentration was used. (B) Interactions of VcPHR and undamaged dsDNA at various protein concentrations. Injection concentrations of 1, 10 and 40 µM were used for VcPHR, while for BSA 10 µM injection concentration was used. Data are mean ±1.4 RU for 3 independent experiments. Response units are statistically significant with p<0.05 unless denoted with*, * = statistically not different.

The amount and the types of damages were defined in previous studies,[31]–[34] in which % 80 of UV light damages on DNA were mentioned to be CPDs, the second abundant lesions were defined as pyrimidine [6–4] pyrimidone photoproduct and small amount of DEWAR photoproducts. Since the most commonly occurring lesion produced by UV light are CPD’s, we decided to use 30 j/m^2^ which was described in previous studies. [29], [35] ssDNA substrate prepared for this study carried mostly CPDs which was in sufficient amount for VcCry and VcPHR to bind and repair in SPR experiments. Double stranded UV damaged DNA was obtained by hybridization of UV damaged single stranded DNA with its unlabelled 48 bp complementary strand in 50 fold excess in hybridization buffer (50 mM Tris-Cl pH7.5 20 mM NaCl). Single stranded 48 bp oligos were mixed and heated at 95°C for 5 minutes. Then the mixture was slowly cooled down to room temperature overnight. This dsDNA substrate was used in appropriate dilutions for SPR assays.

**Figure 4:**
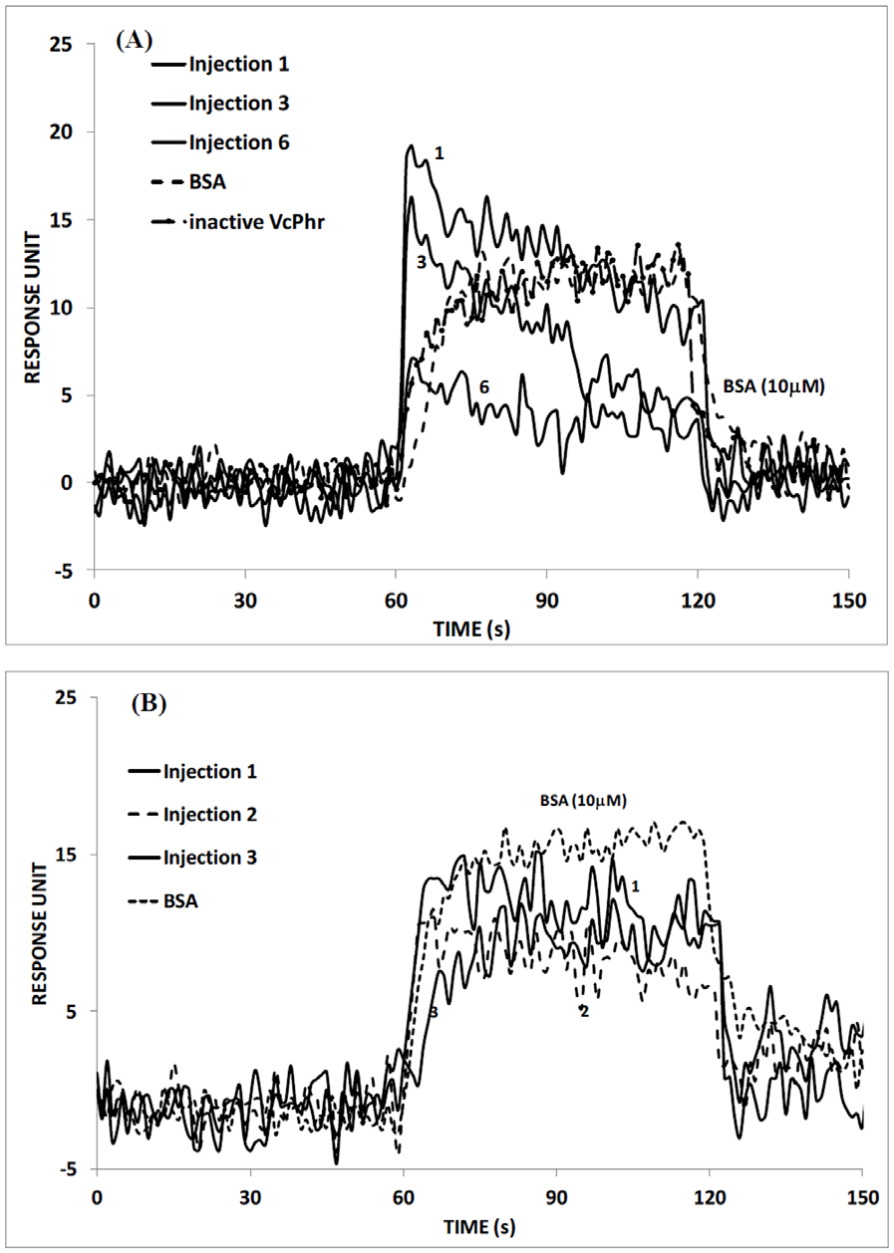
(A) Sequential injection of VcPHR on UV-damaged ssDNA, and injection of inactive VcPHR on UV-damaged ssDNA. (Injection concentration of 1 µM was used for inactive VcPHR.) (B) UV-damaged dsDNA bound surfaces where the concentration of VcPHR was kept constant at 10 nM for all injections. Data are mean ±1.6 RU for 3 independent experiments. Response units are statistically significant with p<0.05.

**Table 1:** Comparison of binding constants obtained from this study and the ones obtained from biochemical assays in the literature.

Protein	SPR Kd	Biochemical Assay
VcPHR/UV damaged ssDNA	0.8–1.8 (±0.3) nM	0.5 nM [27]
VcPHR/Undamaged ssDNA	8.49–22.91 (±1.4) µM	NA
VcPHR/UV damaged dsDNA	18.53–26.08 (±0.5) nM	0.79 nm [45] 4.76 nM [28]
VcPHR/Undamaged dsDNA	8.46–41.30 (±0.6) µM	NA
CRY-DASH/UV damaged ssDNA	29–140 (±7.5) nM	NA
CRY-DASH/Undamaged ssDNA	106.4–128.6(±12.2) µM	NA
CRY-DASH/UV damaged dsDNA	11.6–27.3 (±15.3) µM	NA
CRY-DASH/Undamaged dsDNA	33.8–80.2(±10.7) µM	NA

VcPHR and CRY-DASH have been activated before SPR injections in these experiments.

NA: not available.

### Immobilization of Oligonucleotide Probes

Buffer solutions were freshly prepared from PBS tablets at room temperature just before the experiments started. All buffers were filtered through 0.45 µm filters to reduce particle load and avoid blockages in the instrument. The solution was then degassed using a vacuum pump in order to eliminate the bubbles in the flow cell which may destroy the SPR dips. The pH value of the solution was confirmed as 7.4 with MeterLab® PHM210 standard pH meter. Biotinylated DNA (14 nM) was prepared in PBS solution. Sample volume of 50 µL was used to inject over neutravidin coated surface at a flow rate of 50 µL/min. The system was allowed to equilibrate for 60 minutes.

**Figure 5:**
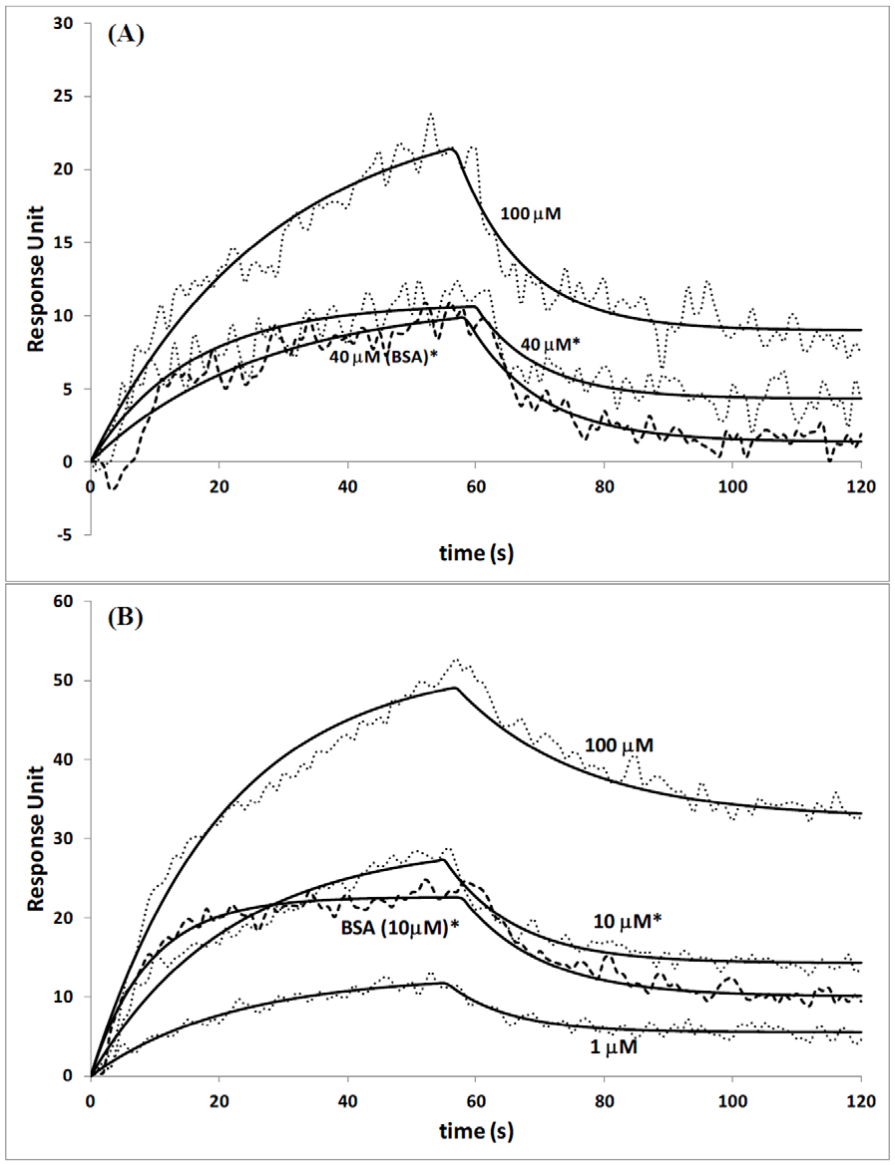
(A) Interactions of CRY-DASH and undamaged ssDNA at various protein concentrations. Injection concentrations of 40, and 100 µM were used for CRY-DASH, while for BSA 40 µM injection concentration was used. (B) Interactions of CRY-DASH and undamaged dsDNA at various protein concentrations. Injection concentrations of 1, 10, and 100 µM were used for CRY-DASH, while for BSA 10 µM injection concentration was used. Data are mean ±2.5 RU for 3 independent experiments. Response units are statistically significant with p<0.05 unless denoted with*, * = statistically not different.

### Assay Design

All protein samples were prepared freshly in 1X PBS solution at pH 7.4 and were activated in 1 mM DTT solution with exposure to blue light at 366 nm wavelength, 2 mW/cm^2^ for 60 minutes. Fifty µL of each sample was injected over the DNA modified surface in the two-channel SPR device for 1 min. Bindings of the proteins to the DNA were recorded as response units. One of the channels was used as a sample channel and the other one was used as a reference in order to the observe the nonspecific interaction in the channel. The details of SPR technique can be found elsewhere. [34] A control experiment was also performed for modified/unmodified-ssDNA/dsDNA in order to see the response of uninteracting couples. BSA is known as an uninteracting protein with ssDNA or dsDNA and was chosen for the control experiments. RU values are plotted after appropriate subtractions were made from a reference surface. Low RU values are measured due to the low surface analyte concentration, which is determined by the concentration of functional groups on the surface. It is also very common to observe RU values within the range of 15–20 in SPR experiments in many different studies. [36]–[38]

**Figure 6:**
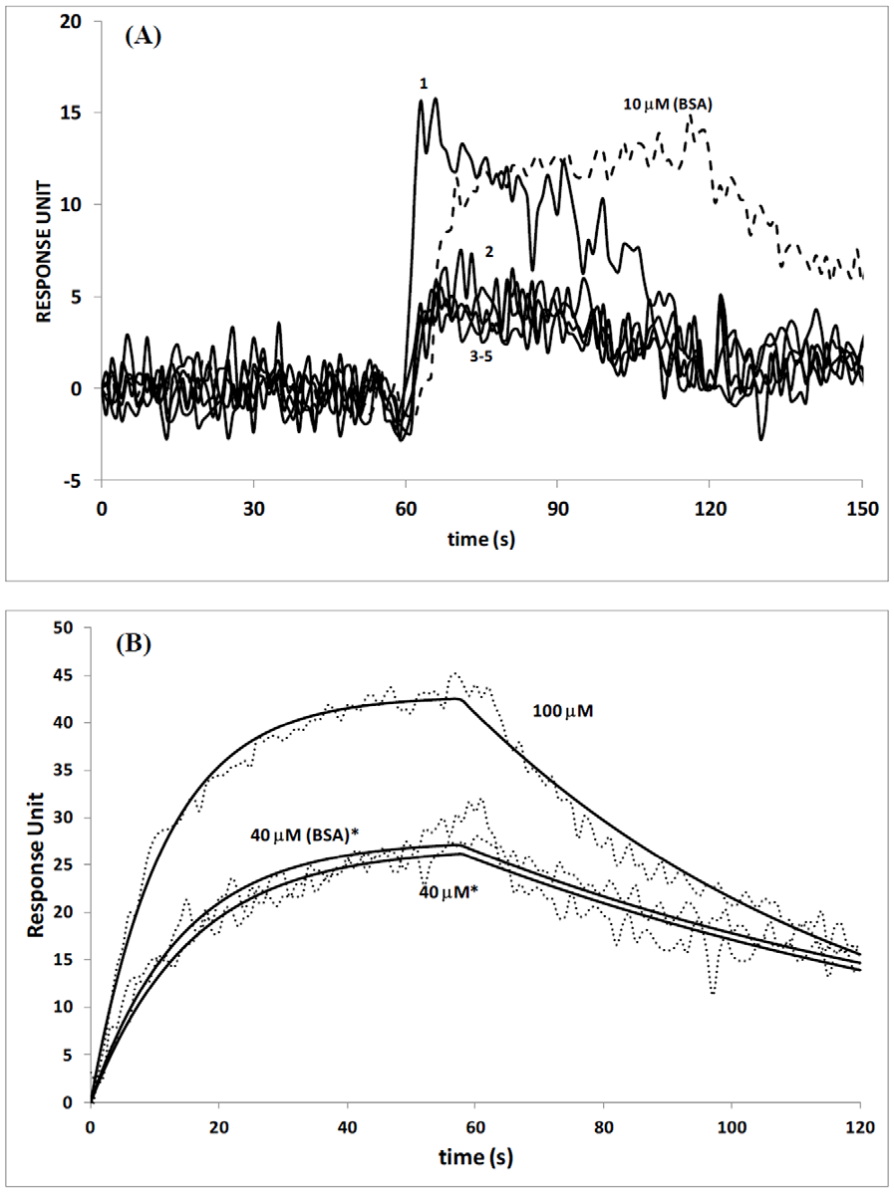
Sequential injection of CRY-DASH on (A) UV-damaged ssDNA, and (B) UV-damaged dsDNA bound surfaces where the concentration of CRY-DASH was kept constant at 10 nM for all injections. Data are mean ±1.7 for 3 independent experiments. Response units are statistically significant with p<0.05 unless denoted with*, * = statistically not different.

### Equilibrium Measurements

Equilibrium analysis helps to determine the strength of binding through two different experiment set ups. Either of the several flowing analyte concentrations are injected until the signal levels out and net association equals to the dissociation. [39] Equilibrium dissociation is reached when the rate of association equals to the rate of dissociation. In other words, dissociation constant, K_D_, is the time required to reach the equilibrium and it could be obtained by plotting the reached response versus the analyte concentration. K_D_ is the equilibrium dissociation constant, which is calculated from the ratio of dissociation constant (k_d_) to the association constant (k_a_):
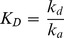
(1)


The response at equilibrium (R_eq_) is close to the maximum response (R_max_), which is reached as the concentration of the analyte becomes greater than K_D_. While performing equilibrium analysis, it is important to use the responses of all analytes at their equilibriums. [39]

### Kinetic Measurements

SPR interactions can be fitted to different models for kinetic analysis; however, it is recommended to use the simplest model. The Langmuir model is one of the most commonly used interaction. It describes 1∶1 interaction in which one ligand molecule interacts with one analyte molecule. This model adapts the pseudo-first-order kinetics and it assumes that the binding is equivalent and independent for all binding sites.

During the first association phase of a biomolecular interaction, the mobile reactant analyte (A) is flowed over the surface of the ligand (L). The concentration of the AL complex modeled in the form of pseudo-first order process. In the second dissociation phase of the complex, a buffer is allowed to flow over the surface to give free analyte and ligand. Let L be the concentration of the ligand and A be the solution concentration of the analyte. The association reaction is as in equation 2:
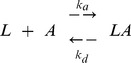
(2)where k_a_ is the association rate constant which describes the rate of complex formation and k_d_ is the dissociation rate constant which describes the stability of the complex. The overall rate law is derived by assuming the order of association reaction and dissociation reactions as first order. The overall rate law is expressed as:



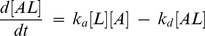
(3)The same equation is applicable to biosensors and with the biosensor response R proportional to AL, equation 3 gives:
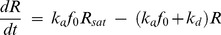
(4)where f_0_ represents L, and R_sat_ represents the response at complete saturation of the immobilized binding sites. If the nonlinear equation is integrated, the following equation will be derived. This type of analysis is applied to reactions which start without mobile reactant bound initially. The time function of binding response is given as:
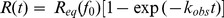
(5)where, the response signal in equilibrium is given by Req as follows:



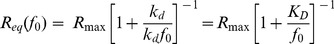
(6)During dissociation phase the free mobile reactant is removed from the buffer, and for the time greater than initial time R(t) is expressed as follows:
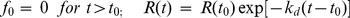
(7)


#### Statistical analysis

The results of all data sets are analyzed using one-way analysis of variance (ANOVA). The results are represented as the mean value (±standard error of mean, SEM) of the triplicate samples unless otherwise stated. The statistical analyses have been done between all data sets in one figure panel. Differences between datasets are considered statistically significant for p-values less than 0.05.

## Results and Discussion

### Purification of Vibrio Cholerae Photolyase and CRY-DASH Proteins

Genes were previously cloned into the pMal-c2x vector to express the corresponding proteins in *E. coli,* fused with maltose binding protein (MBP) to aid in solubility and purification of the recombinant proteins.^26^ Both proteins were expressed at high levels, VcPhr and VcCry1 were soluble and readily purified by affinity chromatography on amylose resin. Figure 1 shows the overexpression and purification of the three proteins by SDS-PAGE. As can be observed in figure 1, the proteins were >95% pure after the affinity purification step and therefore appropriate for further spectroscopic and enzymatic analyses.

Next, proteins were concentrated and checked for the presence of both FAD and MTHF. Figure 2 shows the absorption spectra of VcPhr and VcCry1. The absorption spectrum of VcPhr exhibited a major peak at 380 nm, and minor peaks at 440 and 480 nm, (Figure 2A). This spectrum is consistent with the presence of MTHF, and a mixture of FADH° and FADox in this protein. The near-UV absorption spectrum of VcCry1 was dominated by a peak at 380 nm and, in this preparation, minor absorption beyond 400 nm ascribable to low levels of oxidized forms of flavin (Figure 2B).

### Characterization of DNA Interactions with VcPHR

Figure 3A shows the binding interaction curves between immobilized undamaged ssDNA and VcPHR within a photolyase concentration range of 1–40 µM. In a previous study, Sancar et. al. showed that there was some insignificant binding (<1%) between non-irradiated DNA and photolyase, and that some nonspecific binding was detected only at the highest enzyme concentration. [40] Therefore, contrary to UV-damaged DNA, the concentration of photolyase was increased from nanomolar to micromolar values in order to get a response. As the concentration of the protein increases, the refractive index changes relatively steeper (Figure 3A). However, this bulk index effect were recovered during “the load mode” immediately after the end of injection. A simple pseudo-first order binding interaction model (A+L = AL) which requires only three parameters, i.e. an association rate constant (k_a_), a dissociation rate constant (k_d_) and a maximum surface capacity (R_max_), was fitted using Qdat analysis software. Reference surface data were subtracted from the reaction surface data in order to eliminate the refractive index change. The equilibrium dissociation constant K_D_ (k_d_/k_a_) was found within the range of 8.49–22.91 µM which is quite low, and shows only nonspecific binding between non-irradiated ssDNA and photolyase. A control experiment was also performed with bovine serum albumin (BSA) (10 µM) in order to distinguish the specific interactions from the nonspecific ones. BSA, which has no interactions with ssDNA or dsDNA, [41] was injected over the modified gold surface and the interactions were examined. BSA showed slightly higher interaction with surface bound DNA compared to the response unit observed with 10 µM VcPHR. This result clearly showed nonspecific interaction of tested protein, i.e other components of the sample might be adhering to the sensor surface without a suitable selection of the ligand and that these cannot be attributed to the specific interaction of protein with the surface bound DNA.

Low and nonspecific binding was also observed when VcPHR was injected over undamaged dsDNA bound surfaces (Figure 3B). Compared to unmodified ssDNA, a relatively slower increase in nonspecific binding was observed during injection. However, similar binding intensities (in terms of response units) of VcPHR-dsDNA and VcPHR-ssDNA were observed at the end of the injection phase (15–25 RU). Maximum binding responses in the case of 40 µM VcPHR injection were observed as 22 and 24 RU with dsDNA (Figure 3B) and ssDNA (Figure 3A), respectively. This indicates that the protein has low interaction with unmodified DNA regardless of its form. The SPR analysis showed lower equilibrium dissociation constant, and it was within the range of 8.46–41.30 µM. This is most probably due to the lower affinity of VcPHR to the unmodified dsDNA. It takes longer time to remove the protein from the surface and that the form of the DNA has a slight affect on the dissociation rate.

Next we were interested in exploring the affinity of the both proteins towards their true substrates by SPR. Figure 4A shows the real-time interaction of UV-damaged ssDNA with VcPHR (10 nM in 1X PBS). The numbers on the figure denote injection sequences of VcPHR over UV-damaged DNA bound surface. It is established in the literature that photolyase binds to UV-damaged DNA with high affinity. [42] Photolyase binds to pyrimidine dimers in DNA and accumulation of binding of VcPHR to pyrimidine dimers on the surface increases the refractive index. The immediate increase in response unit right after the beginning of the injection mode could be occurring due to the start of the repair mechanism. As the repair mechanism completes, the photolyase starts to dissociate from the repaired DNA which results in lower refractive index. In this experiment, we have performed 6 consecutive injections of VcPHR onto UV-damaged DNA bound surface. Injections 1–3 showed a decreasing trend in response units even during injection mode, which could be attributed to the continuation of the repair mechanism. The intensity of interaction between the protein (VcPHR) and pyrimidine dimers decreases as the availability of damaged DNA decreases, i.e. as the UV damaged DNA is repaired during a specific injection, lower response units are observed at the subsequent injection. Injection 6 shows the low number of damaged DNA on the surface in the beginning of the experiment. It also shows the completion of the repair in the middle of the injection with a very low response. Injection 6 further confirm that there is no available damage DNA on the surface. In figure 4A, the decrease of the response units during association phase is faster in earlier injections compared to the decreases observed in later injections. This is most probably due to the fast repair of UV damaged DNA during earlier injections which results in fast dissociation in earlier injections. The equilibrium dissociation constant varies within the range of 10^−8^–10^−9 ^M. [43] Our data analysis shows that the K_D_ value varies between 0.8–1.8 nM which is similar to the previously measured biochemical equilibrium dissociation constant of 0.5 nM between VcPHR and UV damaged-ssDNA (Table 1). [43]

In previous studies, it is demonstrated that photolyase had preference for single-stranded over double-stranded substrates. [27], [44] We also studied the interactions between UV-damaged dsDNA and VcPHR (10 nM), and the response curve of the interaction with SPR is shown in Figure 4B. A sudden increase followed by a decrease in the response unit was observed with the start of first injection. Contrary to modified ssDNA, decrease in the response unit was slower when the surface is modified with dsDNA. This may be due to the lower accessibility of the pyrimidine dimers in dsDNA compared to ssDNA. The consecutive injections showed that the repair mechanism continued during the first and the second injections. However, third injection showed a slight increasing trend in response unit which may be an indication of completion of the repair mechanism. The calculated equilibrium dissociation constant found via SPR is within the range of 18.53–26.08 nM and about one order of magnitude higher than the value calculated from the interactions between ssDNA and the VcPHR. The results clearly show that VcPHR bind their substrate with a high affinity, and carry out the desired reaction rapidly.

In order to validate further that specific interactions between UV-damaged ssDNA and VcPHR could be attributed to the DNA repair activity, we investigated the interaction of inactive VcPHR with UV-damaged ssDNA. For this experiment, VcPHR was prepared as inactive by dissolving in PBS only, where the protein was not exposed to DTT and blue light. Figure 4A also demonstrates the real-time interaction of UV-damaged ssDNA with VcPHR (1 µM in 1X PBS). Injection of high concentration of VcPHR (1 µM) onto ssDNA bound surface resulted in low and mostly nonspecific binding. An interesting finding that was obtained in this experiment was the lack of dissociation of the protein from the surface, and hence decreases in response unit during the injection mode. When the active form of the protein was used, the dissociation of the protein from the surface, and decrease in response unit was observed even during injection mode (Figure 4A). The lack of decreasing trend in the response during the injection mode in figure 4A may suggest that the inactive protein, which is incapable of DNA repair, cannot repair UV damaged and surface bound DNA, and hence may be interacting with surface bound DNA in a nonspecific manner.

### Characterization of DNA Interactions with CRY-DASH

Interaction of CRY-DASH was also studied and quantified between ssDNA and dsDNA with SPR (Figure 5). Even though high concentrations of CRY-DASH were used for injections over undamaged ssDNA bound surface, the interactions between the protein and undamaged ssDNA were observed as nonspecific. Injection of high concentration of CRY-DASH onto ssDNA bound surface resulted in very low and mostly nonspecific bindings, where similar responses have been obtained in control experiments done with BSA (Figure 5A). Low affinity for ssDNA also resulted in lower equilibrium dissociation constant, which was calculated within the range of 106.4–128.6 µM. Nonspecific bindings were also observed when high concentrations of CRY-DASH were injected onto dsDNA bound surface (Figure 5B). Even at high concentrations, both BSA and CRY-DASH injections resulted in low intensities in response units.

Selby and Sancar observed that DASH cryptochromes repair CPDs specifically in ssDNA. [24] They were able to observe an apparent binding of *Arabidopsis thaliana* cryptochrome 3 and *Xenopus laevis* XICry-DASH to the dimer in ssDNA. In this study, we investigated the interaction of the CRY-DASH with UV-damaged ssDNA bound surfaces by injecting protein samples over UV-damaged ssDNA bound surface. We performed subsequent five to six separate injections of CRY-DASH onto UV-damaged ssDNA bound surface, where CRY-DASH concentration was kept constant at 10 nM. Despite low concentration of the protein injected, a sudden increase in response curve was observed, while the intensity of the response gradually decreased even during the continuation of the protein injection (Figure 6A). CRY-DASH and UV-damaged ssDNA demonstrated similar interaction profile compared to the one observed with VcPHR and UV-damaged ssDNA. The decreasing trend in response may indicate a repair mechanism on UV-damaged ssDNA by CRY-DASH. Even though subsequent injections did not show significant response changes, the decrease in responses continued by only a few response units. This could be due to the completion of the repair process, where further injections of protein did not result in binding of protein with ssDNA, most probably due to the depletion of damaged sites on surface bound DNA. It is critical to see a decrease in the response curve with the first injection as this decrease in profile shows the dissociation of injected protein from the surface even during injection mode. This could be attributed to real time repair of UV-damaged ssDNA with CRY-DASH. The equilibrium dissociation constant was calculated within the range of 29–140 nM. The equilibrium dissociation constant calculated here is within the nano-molar range, and may be explained by the presence of significant binding interactions between CRY-DASH and UV-damaged ssDNA due to the repair of lesions in DNA structure. Contrary to the interactions observed with UV-damaged ssDNA and CRY-DASH, we did not observe significant binding interactions between UV-damaged dsDNA and CRYDASH (Figure 6B). At higher concentrations of CRYDASH (40 and 100 µM), CRYDASH demonstrated nonspecific interactions with UV-damaged dsDNA, as the trends and magnitude in response units were similar to the binding observed with 40 µm BSA injection (Figure 6B). This could be explained with the absence in repair activity and hence lower affinity of CRYDASH to UV-damaged dsDNA compared to its affinity to UV-damaged ssDNA. Similar result was also observed by Huang et al., where the authors proposed that the free energy of association for Cry-DASH and the CPD lesion is less favorable than the alternative stacking and pairing interactions of the CPD within the dsDNA. [17] As a result, our findings demonstrate that Cry-DASH proteins are unable to stabilize CPD lesions “flipped out” from double strand DNA.

### Conclusions

Interactions and the activity of the photolyase/cryptochrome family group with ss/ds DNA were observed with SPR. The interactions between UV-undamaged-DNA and VcPHR resulted in typical SPR response curves, i.e an increase in the injection mode and a decrease in the load mode. However, the responses observed between UV-damaged-DNA and VcPHR clearly indicated the immediate binding of repair proteins to the target DNA bound to the surface. Decreases in response units observed during injection mode may be explained by the release of proteins just after the completion of the repair of pyrimidine dimers from DNA lesion. The lack of decrease during injection mode for the case of inactive VcPHR injection on ss UV damaged DNA further suggests that repair protein VcPHR specifically interacts with UV-damaged-DNA. Low concentrations of proteins were sufficient to observe the interactions of CRYDASH or VcPHR with UV-damaged ssDNA, where nonspecific interactions were present between UV-damaged dsDNA and CRYDASH or UV damaged ssDNA and VcPHR.

Decrease in binding response profile was observed when CRYDASH was injected onto UV-damaged ssDNA bounds surface. The proteins were able to bind with UV-damaged ssDNA immediately after the start of injection mode, which was an indication of specific interactions between proteins and DNA, and high tendency to repair the pyrimidine dimers. The release of proteins from DNA bound surface was probably due to the completion of proteins’ task of repairing lesions. Unlike the specific interactions observed with UV-damaged ssDNA and CRYDASH, we did not observe specific interactions between CRYDASH and UV-damaged dsDNA, which confirms the previous findings about the interactions of this protein and UV-damaged dsDNA.

These results clearly depict the effective use of SPR in order to see the real time response of the interacting molecules, i.e protein and modified-DNA in this study. The technique requires only small amounts of the samples to be tested. The binding parameters were determined by the analysis of the association and dissociation phases. The strength of association and the tendency of dissociation during the injection mode most probably represent the real time repair mechanism. Our result support separate biochemical assay findings in the literature, where SPR was used here to quantify and demonstrate the interactions of UV damaged/undamaged ss/dsDNA with flavoproteins. The comparison of binding interactions with the active and inactive repair protein VcPHR and UV damaged ssDNA further suggest that it might be possible to monitor repair activities of these proteins using SPR.

## Supporting Information

Figure S1 **Formation of CPDs as a function of UV exposure time.**
(TIF)

## References

[1] Weber S (2005) Light-driven enzymatic catalysis of DNA repair: a review of recent biophysical studies on photolyase. Biochimica et Biophysica Acta (BBA) - Bioenergetics 1707: 1–23. 15721603 10.1016/j.bbabio.2004.02.010

[2] Donahue BA, Yin S, Taylor JS, Reines D, Hanawalt PC (1994) Transcript cleavage by RNA polymerase II arrested by a cyclobutane pyrimidine dimer in the DNA template. Proc Natl Acad Sci U S A 91: 8502–8506. 8078911 10.1073/pnas.91.18.8502 PMC44634

[3] Hanawalt PC (1994) Transcription-coupled repair and human disease. Science 266: 1957–1958. 7801121 10.1126/science.7801121

[4] Otoshi E, Yagi T, Mori T, Matsunaga T, Nikaido O, et al (2000) Respective roles of cyclobutane pyrimidine dimers, (6–4)photoproducts, and minor photoproducts in ultraviolet mutagenesis of repair-deficient xeroderma pigmentosum A cells. Cancer Res 60: 1729–1735. 10749146

[5] Heelis PF, Kim ST, Okamura T, Sancar A (1993) New trends in photobiology: The photo repair of pyrimidine dimers by DNA photolyase and model systems. Journal of Photochemistry and Photobiology B: Biology 17: 219–228. 10.1016/1011-1344(93)80019-6 8492239

[6] Okamura T, Sancar A, Paul F (1991) Picosecond Laser Photolysis Studies on the Photorepair of Pyrimidine Dimers by DNA Photolyase. 1. Laser Photolysis of Photolyase-2-Deoxyuridine Dinucleotide Photodimer Complex. J Am Chem Soc 113: 3143–3145.

[7] Sancar A (2003) Structure and function of DNA photolyase and cryptochrome blue-light photoreceptors. Chemical Reviews 103: 2203–2237. 12797829 10.1021/cr0204348

[8] Ozturk N, Selby CP, Annayev Y, Zhong D, Sancar A (2011) Reaction mechanism of Drosophila cryptochrome. PNAS 108: 516–521. 21187431 10.1073/pnas.1017093108 PMC3021015

[9] Kavakli IH, Sancar A (2002) Circadian photoreception in humans and mice. Mol Interventions 2: 484–492. 10.1124/mi.2.8.484 14993400

[10] Cashmore AR, Jarillo JA, Wu YJ, Liu D (1999) Cryptochromes: blue light receptors for plants and animals. Science 284: 760–765. 10221900 10.1126/science.284.5415.760

[11] Sancar A, Thompson C, Thresher R, Araujo F, Mo J, et al (2000) Photolyase/cryptochrome family blue-light photoreceptors use light energy to repair DNA or set the circadian clock. Cold Spring Harb Symp Quant Biol 65: 157–171. 12760030 10.1101/sqb.2000.65.157

[12] Li QH, Yang HQ (2007) Cryptochrome signaling in plants. Photochem Photobiol 83: 94–101. 17002522 10.1562/2006-02-28-IR-826

[13] van der Horst GT, Muijtjens M, Kobayashi K, Takano R, Kanno S, et al (1999) Mammalian Cry1 and Cry2 are essential for maintenance of circadian rhythms. Nature 398: 627–630. 10217146 10.1038/19323

[14] Daiyasu H, Ishikawa T, Kuma K, Iwai S, Todo T, et al (2004) Identification of cryptochrome DASH from vertebrates. Genes Cells 9: 479–495. 15147276 10.1111/j.1356-9597.2004.00738.x

[15] Kleine T, Lockhart P, Batschauer A (2003) An Arabidopsis protein closely related to Synechocystis cryptochrome is targeted to organelles. Plant J 35: 93–103. 12834405 10.1046/j.1365-313x.2003.01787.x

[16] Brudler R, Hitomi K, Daiyasu H, Toh H, Kucho K, et al (2003) Identification of a new cryptochrome class. Structure, function, and evolution. Mol Cell 11: 59–67. 12535521 10.1016/s1097-2765(03)00008-x

[17] Huang Y, Baxter R, Smith BS, Partch CL, Colbert CL, et al (2006) Crystal structure of cryptochrome 3 from Arabidopsis thaliana and its implications for photolyase activity. Proc Natl Acad Sci U S A 103: 17701–17706. 17101984 10.1073/pnas.0608554103 PMC1635974

[18] Komori H, Masui R, Kuramitsu S, Yokoyama S, Shibata T, et al (2001) Crystal structure of thermostable DNA photolyase: pyrimidine-dimer recognition mechanism. Proc Natl Acad Sci U S A 98: 13560–13565. 11707580 10.1073/pnas.241371398 PMC61080

[19] Park HW, Kim ST, Sancar A, Deisenhofer J (1995) Crystal structure of DNA photolyase from Escherichia coli. Science 268: 1866–1872. 7604260 10.1126/science.7604260

[20] Brautigam CA, Smith BS, Ma Z, Palnitkar M, Tomchick DR, et al (2004) Structure of the photolyase-like domain of cryptochrome 1 from Arabidopsis thaliana. Proc Natl Acad Sci U S A 101: 12142–12147. 15299148 10.1073/pnas.0404851101 PMC514401

[21] Mees A, Klar T, Gnau P, Hennecke U, Eker AP, et al (2004) Crystal structure of a photolyase bound to a CPD-like DNA lesion after in situ repair. Science 306: 1789–1793. 15576622 10.1126/science.1101598

[22] Saxena C, Wang H, Kavakli IH, Sancar A, Zhong D (2005) Ultrafast dynamics of resonance energy transfer in cryptochrome. J Am Chem Soc 127 7984–7985. 10.1021/ja0421607 15926801

[23] Lin C, Todo T (2005) The cryptochromes. Genome Biology 6: 220. 15892880 10.1186/gb-2005-6-5-220 PMC1175950

[24] Selby CP, Sancar A (2006) A cryptochrome/photolyase class of enzymes with single-stranded DNA-specific photolyase activity. Proc Natl Acad Sci USA 103: 17696–17700. 17062752 10.1073/pnas.0607993103 PMC1621107

[25] Sancar A (2003) Structure and function of DNA photolyase and cryptochrome bluelight photoreceptors. Chem Rev 103 2203–2237. 10.1021/cr0204348 12797829

[26] Swinney DC (2004) Biochemical mechanisms of drug action: what does it take for success? Nat Rev Drug Discov 3: 801–808. 15340390 10.1038/nrd1500

[27] Zhao XD, Liu JQ, Hsu DS, Zhao SY, Taylor JS, et al (1997) Reaction mechanism of (6–4) photolyase. Journal of Biological Chemistry 272: 32580–32590. 9405473 10.1074/jbc.272.51.32580

[28] Hitomi K, Kim ST, Iwai S, Harima N, Otoshi E, et al (1997) Binding and catalytic properties of Xenopus (6–4) photolyase. Journal of Biological Chemistry 272: 32591–32598. 9405474 10.1074/jbc.272.51.32591

[29] Worthington EN, Kavakli IH, Berrocal-Tito G, Bondo BE, Sancar A (2003) Purification and characterization of three members of the photolyase/cryptochrome family blue-light photoreceptors from Vibrio cholerae. Journal of Biological Chemistry 278: 39143–39154. 12878596 10.1074/jbc.M305792200

[30] Smith CA, Taylor SJ (1993) Preparation and Characterization of a Set of Deoxyoligonucleotide 49- mers Containing Site-specific Cis-syn, Trans-syn-I(,6–4), and Dewar Photoproducts of Thymidylyl(3′ +5′)-thymidine. J Biol Chem 268: 11143–11151. 8496175

[31] Pfeifer GP (1997) Formation and processing of UV photoproducts: Effects of DNA sequence and matin envirnoment. Photochem Photobiol 65: 270–283. 9066304 10.1111/j.1751-1097.1997.tb08560.x

[32] Svoboda LD, Smith CA, Taylor JS, Sancar A (1993) Effect of sequence, adduct type and the opposing lesions on the binding and repair of ultraviolet photodamage by DNA photolyase and (A)BC excinuclease. J Biol Chem 268: 10694–10700. 8486719

[33] You Y-H, Lee D-H, Yoon J-H, Nakajima S, Yasui A, et al (2001) Cyclobutane pyrimidine dimers are responsible for the vast majority of mutations induced by UVB irradiation in mammalian cells. J Biol Chem 276: 44688–44694. 11572873 10.1074/jbc.M107696200

[34] Liu Y, Wilson WD (2010) New York, NY: Humana Press. 1–23. p.

[35] Asimgil H, Kavakli IH (2012) Purification and Characterization of five members of photolayse/cryptochrome family from Cyanidioschyzon merolae. Plant Sci 185–186: 190–198. 10.1016/j.plantsci.2011.10.005 22325881

[36] Rich RL, Myszka DG (2000) Advances in surface plasmon resonance biosensor analysis. Current Opinion in Biotechnology 11: 54–61. 10679342 10.1016/s0958-1669(99)00054-3

[37] Day YSN, Myszka DG (2003) Characterizing a drug’s primary binding site on albumin. Journal of Pharmaceutical Sciences 92: 333–343. 12532383 10.1002/jps.10293

[38] Henn C, Boettcher S, Steinbach A, Hartmann RW (2012) Catalytic enzyme activity on a biosensor chip: Combination of surface plasmon resonance and mass spectrometry. Analytical Biochemistry. 10.1016/j.ab.2012.05.024 22677626

[39] Roos H, Karlsson R, Nilshans H, Persson A (1998) Thermodynamic analysis of protein interactions with biosensor technology. Journal of Molecular Recognition 11: 204–210. 10076841 10.1002/(SICI)1099-1352(199812)11:1/6<204::AID-JMR424>3.0.CO;2-T

[40] Sancar GB, Smith FW, Sancar A (1985) Binding of Escherichia coli DNA Photolyase to UV-Irradiated DNA. Biochemistry 24: 1849–1855. 3893538 10.1021/bi00329a007

[41] Boon EM, Salas JE, Barton JK (2002) An electrical probe of protein-DNA interactions on DNA-modified surfaces. Nature Biotechnology 20: 282–286. 10.1038/nbt0302-282 11875430

[42] Fox ME, Feldman BJ, Chu G (1994) A Novel Role for DNA Photolyase - Binding to DNA Damaged by Drugs Is Associated with Enhanced Cytotoxicity in Saccharomyces-Cerevisiae. Molecular and Cellular Biology 14: 8071–8077. 7969145 10.1128/mcb.14.12.8071-8077.1994 PMC359345

[43] Yang K, Matsika S, Stanley RJ (2007) 6MAP, a Fluorescent Adenine Analogue, Is a Probe of Base Flipping by DNA Photolyase. J Phys Chem B 111: 10615–10625. 17696385 10.1021/jp071035p

[44] Husain I, Sancar GB, Holbrook SR, Sancar A (1987) Mechanism of Damage Recognition by Escherichia-Coli DNA Photolyase. Journal of Biological Chemistry 262: 13188–13197. 3308872

[45] Husain I, Sancar A (1987) Binding of E. coli DNA photolyase to a defined substrate containing a single T mean value of T dimer. Nucleic Acids Res 15: 1109–1120. 3547332 10.1093/nar/15.3.1109 PMC340511

