# Correction: Investigation of Real-Time Photorepair Activity on DNA via Surface Plasmon Resonance

**DOI:** 10.1371/annotation/759813e6-647e-4d4a-be5e-c022281960f1

**Published:** 2012-10-25

**Authors:** Rıza Kizilel, Enis Demir, Selimcan Azizoglu, Hande Asimgil, Ibrahim Halil Kavakli, Seda Kizilel

The fourth author's name was spelled incorrectly. The correct name is: Hande Asimgil.

The correct citation is: Kizilel R, Demir E, Azizoglu S, Asimgil H, Kavakli IH, et al. (2012) Investigation of Real-Time Photorepair Activity on DNA via Surface Plasmon Resonance. PLoS ONE 7(8): e44392. doi:10.1371/journal.pone.0044392 

