# Supplementary figures and images for: Investigation of Real-Time Photorepair Activity on DNA via Surface Plasmon Resonance
Source: PLoS One. 2012 Aug 29;7(8):e44392. doi: 10.1371/journal.pone.0044392 (PMC3430658; doi:10.1371/journal.pone.0044392)

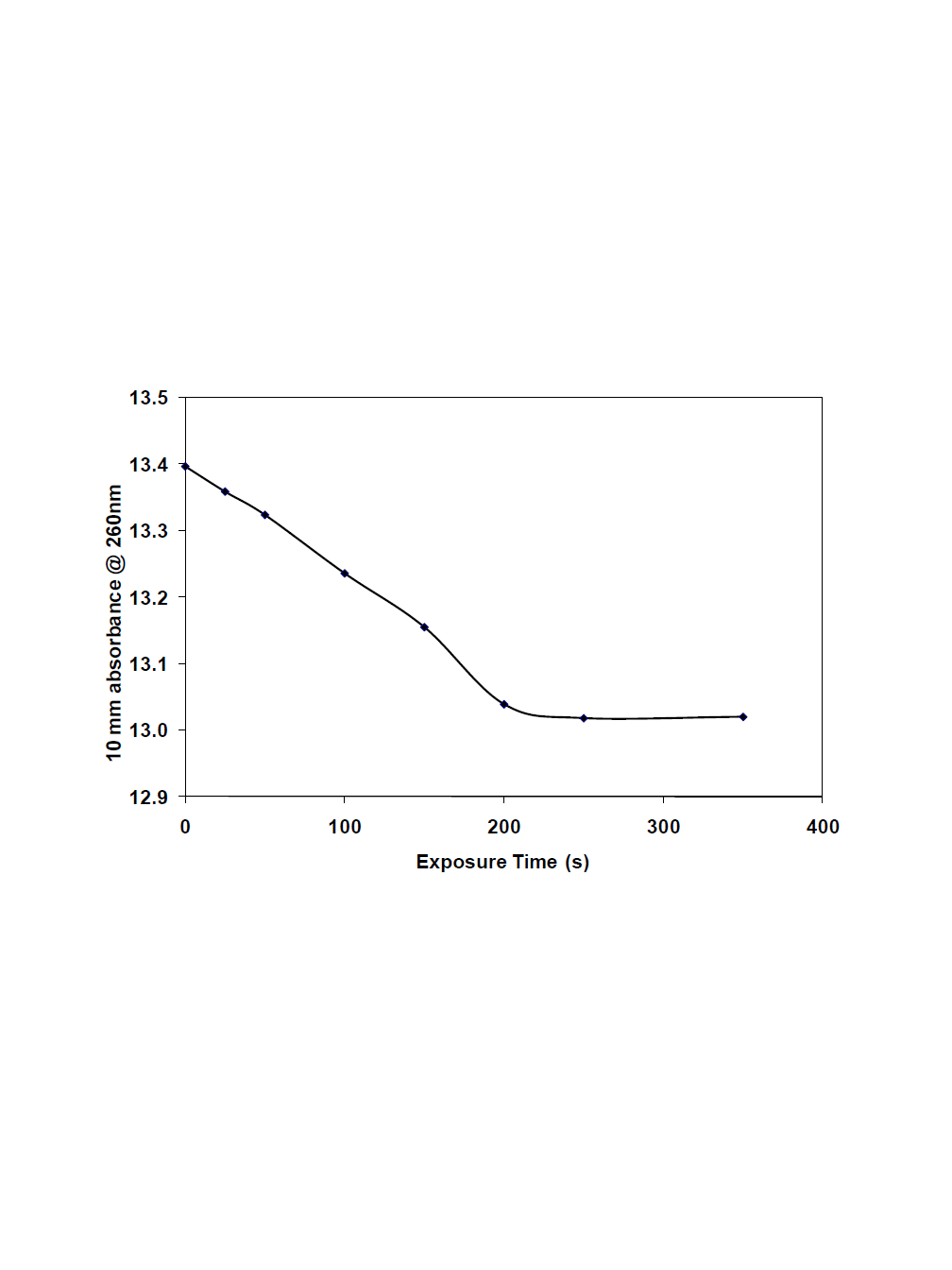

Supplement: Figure S1 — Formation of CPDs as a function of UV exposure time. (TIF) [file pone.0044392.s001.tif]
